# The Treatment of Gonorrhea

**Published:** 1903-12

**Authors:** S. D. Warnock

**Affiliations:** Atlanta, Ga.


### THE TREATMENT OF GONORRHEA.

BY S. D. WARNOCK, M.D.,
ATLANTA, GA.

Recognizing that there is "nothing new under the sun," it is with some little hesitancy that I undertake to write a few words upon the practical treatment of gonorrhea. Much has been written upon this subject, but we are still confronted with the problem of a cure for this disease. It would seem that a specific was out of the question, but the physician of to-day has at his disposal means that, judiciously used, unquestionably would prove satisfactory in the vast majority of cases. The trouble has been that the physician could not exercise his knowledge at a time when it was most useful, namely, in the early stages. However, the laity are beginning to appreciate that gonorrhea can no longer be classed as a mere cold or strain, and having been enlightened as to its seriousness, especially as to the many possible complications, they are beginning to consult the physician at an earlier period and not after the disease has become complicated through mistreatment and neglect.

It is an established fact that gonorrhea is due to a specific organism which must be destroyed before a permanent cure can be obtained, and for this purpose a large number of drugs have been recommended. It is not my object here to enter into a discussion of their relative value, for, as we all know, one observer obtains results with a certain remedy which can not be duplicated by another. The cause of these discrepancies in the practice of careful and trustworthy observers can only be sought in the method of treatment and application of the drug, and hence this is of particular importance.

During the past two years I have treated many cases of acute and chronic urethritis in the following manner: A three-drachm syringe is filled with a solution of protargol, one and one-half grains to the ounce; the patient injects this at my office and retains it for a few minutes. This is done daily for one week, increasing one grain per ounce each day. The second week the patient is told to return every other day, and the injection is increased one grain per ounce at each visit. This is continued until the strength of the solution has reached nine grains to the ounce. Then it is used twice a week until the urine is free from shreds, which usually takes place in five or six weeks of treatment. For that class of patients who cannot make a daily visit to my office, I give them a solution containing one grain of protargol to the ounce, with instructions to inject three times a day after urinating; when the patient returns for the next bottle I order two grains to the ounce, and so on, until nine grains to the ounce; then it is used twice a week.

In making solutions of protargol only cold water should be used, as it has been shown that heat effects a certain amount of decomposition and may render the injection more or less irritating.

The subsequent use of astringents is not necessary. This treatment has been devoid of irritation and discomfort, and I have not found it necessary to employ cocain even in cases in which the mucous membrane is very sensitive.

Of course the condition of the bowels should be looked after, and should constipation exist a saline laxative, as citrate of magnesia in the effervescent form, is prescribed. If the urethra is especially sensitive it is best to make the urine decidedly alkaline.

As to the frequency of complications, none were observed in all cases seen early in the disease in which the anterior urethra was alone affected; in those cases in which the posterior urethra was involved epididymitis occurred in fifteen per cent. of the cases.

If the patient is in the chronic stage of the disease I direct him to call at my office daily, and after letting him urinate, I wash out the urethra with cold water, and then employ deep injections of a one per cent. solution of protargol, gradually increased to four or five per cent. He is also given a one or two per cent. solution to use in the interval twice daily. Both in the acute and chronic stages I make it a practice to examine carefully the urethral discharge for gonococci, as this affords the only safe criterion by which to judge whether the disease has been radically removed.

In conclusion, I would say that I have had far better results with the treatment outlined above than with any other I have ever used.

---

AT the recent meeting of the American Public Health Association held at Washington the committee on vital statistics reported that effective co-operation had been instituted between that Association, the Conference of State Boards of Health, the American Medical Association, the United States Census Bureau and the United States Public Health and Marine-Hospital Service for the improvement of the vital statistics of this country. Among the objects sought are the extension of adequate methods of registration, the use of uniform and comparable tables and rates in bulletins and reports, and the improvement of the international classification of causes of death. A pamphlet on "Statistical Treatment of Causes of Death" has been issued by the United States Census Bureau, requests for which should be addressed to Mr. W. A. King, Chief Statistician for Vital Statistics, Census Bureau. It has special reference to the difficulties encountered in compiling deaths returned from several causes, and asks for the co-operation of the profession in framing a thoroughly satisfactory method of procedure in such cases.

